# New Derivatives of Pyridoxine Exhibit High Antibacterial Activity against Biofilm-Embedded* Staphylococcus* Cells

**DOI:** 10.1155/2015/890968

**Published:** 2015-12-29

**Authors:** Airat R. Kayumov, Aliya A. Nureeva, Elena Yu. Trizna, Guzel R. Gazizova, Mikhail I. Bogachev, Nikita V. Shtyrlin, Mikhail V. Pugachev, Sergey V. Sapozhnikov, Yurii G. Shtyrlin

**Affiliations:** ^1^Kazan Federal University, Kremlevskaya Street 18, Kazan 420008, Russia; ^2^Biomedical Engineering Research Center, Saint Petersburg Electrotechnical University, Professor Popov Street 5, Saint Petersburg 197376, Russia

## Abstract

Opportunistic bacteria* Staphylococcus aureus *and* Staphylococcus epidermidis* often form rigid biofilms on tissues and inorganic surfaces. In the biofilm bacterial cells are embedded in a self-produced polysaccharide matrix and thereby are inaccessible to biocides, antibiotics, or host immune system. Here we show the antibacterial activity of newly synthesized cationic biocides, the quaternary ammonium, and bisphosphonium salts of pyridoxine (vitamin B6) against biofilm-embedded* Staphylococci*. The derivatives of 6-hydroxymethylpyridoxine were ineffective against biofilm-embedded* S. aureus *and* S. epidermidis* at concentrations up to 64 *μ*g/mL, although all compounds tested exhibited low MICs (2 *μ*g/mL) against planktonic cells. In contrast, the quaternary ammonium salt of pyridoxine (N,N-dimethyl-N-((2,2,8-trimethyl-4H-[1,3]dioxino[4,5-c]pyridin-5-yl)methyl)octadecan-1-aminium chloride (**3**)) demonstrated high biocidal activity against both planktonic and biofilm-embedded bacteria. Thus, the complete death of biofilm-embedded* S. aureus* and* S. epidermidis* cells was obtained at concentrations of 64 and 16 *μ*g/mL, respectively. We suggest that the quaternary ammonium salts of pyridoxine are perspective to design new synthetic antibiotics and disinfectants for external application against biofilm-embedded cells.

## 1. Introduction

Opportunistic bacteria like* Escherichia coli*,* Micrococcus *sp.,* Staphylococcus *sp., and others have been demonstrated to form rigid biofilms, a community of microbial cells adherent to a substrate and embedded in a polysaccharide matrix (EPS) produced by cells themselves [1]. Being in biofilm, bacteria are extremely resistant to biocides, antibiotics and to the human immune system [1, 2]. This leads to the drastic increase in the bacterial resistance against antimicrobial treatment, higher frequency of nosocomial infections, and creating difficulties in the microbiological diagnostics of infectious diseases [2, 3].* Staphylococcus aureus *and* Staphylococcus epidermidis* often form a rugged biofilm on surfaces of medical devices, catheters, and plastic implants causing chronic reinfections and inflammatory complications during the postoperative period [3–5]. Accordingly the efficacy of currently commercially available antibiotics is severely reduced in the presence of such biofilms and the development of new antimicrobial biofilm agents that could overcome this limitation is one of the important challenges in pharmaceutical industry.

Several approaches offered to date could resolve this problem, like (a) biofilm destruction, (b) biofilm formation inhibition, and (c) antimicrobials diffusing into the biofilm (for an extensive review, we refer to [6, 7] and references therein). Thus, some proteases and nucleases were shown to destroy the biofilm backbone and to enhance the efficiency of antimicrobials [8, 9]. In particular, the glycosidase pectinase and the protease subtilisin A have been shown to enhance* Escherichia coli* sensitivity to ampicillin [10]. Additionally, the biofilm formation could be blocked by either natural agents like c-di-AMP or synthetic compounds like furanones that affect quorum sensing [11–14]. Nevertheless, in the above examples only prevention or disruption of the biofilm occurs and combining with additional antimicrobial treatment is required [6]. Therefore development of antimicrobials that are able either to diffuse or to be delivered into bacterial biofilms seems to have considerable benefits. However until now very few antibiotics that are able to penetrate into biofilms themselves have been reported. For example, delafloxacin was shown to diffuse into* S. aureus* exopolysaccharide matrix [15], while tetracycline and daptomycin quickly moved into* Escherichia coli* and* Staphylococcus epidermidis* biofilms [16, 17]. Alternatively, lipid and polymer nanoparticles were found to increase the antimicrobial efficacy in many cases (for an extensive review, we refer to [18] and references therein).

Cationic surfactants have been widely adopted as antiseptics and disinfectants for a variety of clinical purposes such as preoperative disinfection of the intact skin, application to mucous membranes, disinfection of noncritical surfaces, and many other applications [19, 20]. Among them, the quaternary ammonium salts were shown to be highly effective against gram-positive bacteria including* S. aureus *and* S. epidermidis* (reviewed in [21]). In combination with silver nanoparticles, the quaternary ammonium salts have demonstrated high efficiency against microorganisms located in biofilms [22, 23]. Several investigations indicate that the cationic part of quaternary ammonium salts seems to be responsible for diffusion into the biofilm and this way for the drug delivery [21].

In our previous works we reported for the first time the synthesis of cationic biocides series (quaternary ammonium and phosphonium salts) based on pyridoxine (vitamin B6) [24–26]. Some of these compounds demonstrated high antibacterial activity against planktonic cells of* Staphylococcus aureus *and* Staphylococcus epidermidis* multidrug resistant clinical isolates [24, 25]. In these papers the relationship between the location of quaternary ammonium and phosphonium fragments in the pyridoxine molecule and the antibacterial activity, lipophilicity, and toxicity of the compound is shown. Our aim here was to study the biocidal activity of these compounds against biofilm-embedded* Staphylococcus* cells as well. Using the drop plate method and the differential fluorescent microscopy to estimate the viability of bacteria, we show explicitly that, in contrast to ciprofloxacin, the quaternary ammonium salt of pyridoxine (N,N-dimethyl-N-((2,2,8-trimethyl-4H-[1,3]dioxino[4,5-c]pyridin-5-yl)methyl)octadecan-1-aminium chloride) completely kills the biofilm-embedded* S. aureus* and* S. epidermidis* cells at concentrations of 64 and 16 *μ*g/mL, respectively. Therefore, the quaternary ammonium salts of pyridoxine derivatives seem to be perspective candidates for the design of new synthetic antimicrobials, due to their efficiency against the biofilm-embedded cells.

## 2. Materials and Methods

**Figure 1:**
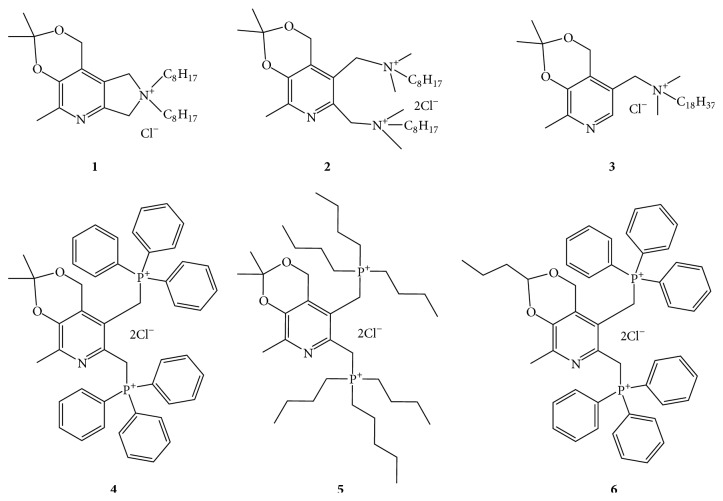
Structures of pyridoxine derivatives used in this study:** 1**: 3,3,5-trimethyl-8,8-dioctyl-1,7,8,9-tetrahydro-[1,3]dioxino[5,4-d]pyrrolo[3,4-b]pyridin-8-ium chloride;** 2**: N,N′-((2,2,8-trimethyl-4H-[1,3]dioxino[4,5-c]pyridine-5,6-diyl)bis(methylene))bis(N,N-dimethyloctan-1-aminium) dichloride;** 3**: N,N-dimethyl-N-((2,2,8-trimethyl-4H-[1,3]dioxino[4,5-c]pyridin-5-yl)methyl)octadecan-1-aminium chloride;** 4**: 5,6-bis(triphenylphosphonio(methyl))-2,2,8-trimethyl-4H-[1,3]dioxino[4,5-c]pyridine dichloride;** 5**: 5,6-bis[(tributylphosphonio)methyl]-2,2,8-trimethyl-4H-[1,3]dioxino[4,5-c]pyridine dichloride;** 6**: 5,6-bis[triphenylphosphonio(methyl)]-8-methyl-2-propyl-4H-[1,3]dioxino[4,5-c]pyridine dichloride.

Figure 1 shows the structures of the quaternary ammonium (compounds** 1**–**3**) and of the bisphosphonium (compounds** 4**–**6**) salts of pyridoxine and 6-hydroxymethylpyridoxine; compounds were synthesized and described previously [24–26]:** 1**: 3,3,5-trimethyl-8,8-dioctyl-1,7,8,9-tetrahydro-[1,3]dioxino[5,4-d]pyrrolo[3,4-b]pyridin-8-ium chloride;** 2**: N,N′-((2,2,8-trimethyl-4H-[1,3]dioxino[4,5-c]pyridine-5,6-diyl)bis(methylene))bis(N,N-dimethyloctan-1-aminium) dichloride;** 3**: N,N-dimethyl-N-((2,2,8-trimethyl-4H-[1,3]dioxino[4,5-c]pyridin-5-yl)methyl)octadecan-1-aminium chloride;** 4**: 5,6-bis(triphenylphosphonio(methyl))-2,2,8-trimethyl-4H-[1,3]dioxino[4,5-c]pyridine dichloride;** 5**: 5,6-bis[(tributylphosphonio)methyl]-2,2,8-trimethyl-4H-[1,3]dioxino[4,5-c]pyridine dichloride;** 6**: 5,6-bis[triphenylphosphonio(methyl)]-8-methyl-2-propyl-4H-[1,3]dioxino[4,5-c]pyridine dichloride.

Ciprofloxacin (Sigma), vancomycin (Sigma), and miramistin (Infamed, Russia) were used as reference antibiotics.

### 2.1. Strains and Culture Conditions

The following strains were used in the study:* Staphylococcus aureus *subsp.* aureus* (ATCC 29213) and* Staphylococcus epidermidis *(clinical isolate obtained from the Kazan Institute of Epidemiology and Microbiology, Kazan, Russia).

All the bacterial strains were maintained and cultured in a LB medium (10.0 g/L of tripton; 5 g/L of yeast extract; 5 g/L of NaCl; pH 8.5) [27]. The Mueller-Hinton broth (Fluka), Trypticase soy broth (Sigma), Luria-Bertani broth (LB) (Trytone 10.0 g, yeast extract 5.0 g, and NaCl 5.0 g in 1.0 liter tap water), and modified Basal medium (BM) (glucose 5.0 g, peptone 7.0 g, MgSO_4_  × 7H_2_O 2.0 g, and CaCl_2_ 0.05 g in 1.0 liter tap water) were used as indicated. For the biofilm assays, bacteria were grown in BM for 72 h without shaking at 37°C to obtain rigid biofilms [13, 28].

### 2.2. Biofilm Staining with Crystal Violet

Biofilm formation was assessed either in 35 mm polystyrol tissue culture treated plates (Eppendorf) or in 96-well polystyrol tissue culture treated plates (Eppendorf) and analyzed with crystal violet staining as described previously [29]. Bacteria were cultured in 2 mL of BM at 37°С without shaking with an initial density of 3 × 10^7^ CFU/mL. After 72 h of incubation, the culture liquid was removed; the plates were washed twice with phosphate-buffered saline (PBS) pH 7.4 and dried for 20 min. Then either 1 or 0.2 mL of 0.2% crystal violet solution (Sigma) in 96% ethanol was added per plate or well, respectively, followed by 20 min incubation. Next the crystal violet solution was removed and the plate was washed 3 times with PBS. After 30 min air drying, 1 mL of 96% ethanol was added to resolubilize bound crystal violet, and the absorbance was read at 570 nm with the microplate reader Tecan Infinite 200 Pro. Cell-free wells incubated with pure medium subjected to all staining manipulations were used as a control.

### 2.3. Evaluation of Antibacterial Activity

The minimum inhibitory concentration (MIC) of compounds was determined by the broth microdilution method in both Mueller-Hinton medium and BM in 96-well nontreated cell culture plates (Eppendorf). The concentrations of antimicrobials after serial 2-fold dilutions were in the range of 0.5–512 *µ*g/mL. Wells were seeded with 200 *µ*L of the bacterial culture (3 × 10^7^ CFU/mL) and incubated at 37°C. The MIC was determined as the lowest concentration of compound for which no visible bacterial growth could be observed after 24 h of incubation. To determine the minimum bactericidal concentration (MBC), 5 *μ*L of culture liquid from wells with no visible growth was inoculated into 5 mL of LB broth and cultivated for 24 h. The MBC was determined as the lowest concentration of compound for which no visible bacterial growth could be observed.

To evaluate the antibacterial activity against biofilm-embedded cells, rigid biofilms have been preformed (72 h growth in BM broth) and the plates were washed twice with sterile broth, followed by the exchange of the old medium by the new one. The compounds of interest were added as indicated and the incubation was continued for the next 24 h [30].

The viability of the detached cells in culture liquid was evaluated by the drop plate method [31]. The serial 10-fold dilutions of each well were prepared and 10 *μ*L of suspension was dropped onto LB plates. CFUs were counted from those drops containing 5–10 colonies.

To evaluate the viability of biofilm-embedded cells by differential fluorescent microscopy, wells were washed several times with phosphate-buffered saline (PBS) to remove nonadherent and detached cells. The residual buffer was removed by pipetting and the staining solution (5 *μ*g/mL acridine orange and 3 *μ*g/mL propidium iodide (Sigma) in PBS [32, 33]) was loaded for 10 min. Next the liquid was removed; cells were washed once by PBS and analyzed with immersion on fluorescent Karl Zeiss Axio 2.0 microscope. Both green and red fluorescent microphotographs (all cells and dead cells, resp.) of each field of view were obtained followed by overlaying of the green images by the red images. Alternatively, the washed biofilms were suspended in PBS by scratching the well bottoms with following treatment in a sonicator bath for 2 min to favor the disintegration of bacterial clumps [30], and viable cells were counted by the drop plate method [31] as described above. MBC^adh^ was defined as the lowest concentration of antibiotic leading to nonviable cells in adherent microcolonies being detected [34, 35].

### 2.4. Statistics

All experiments were performed in biological triplicate with three repeats in each one. Data from biofilm crystal violet staining were compared against control using the Wilcoxon signed-rank test (paired difference test). Differences were considered significant at *P* < 0.05. The fraction of nonviable cells was estimated as the relative number of the red cells in the combined images obtained by overlaying of the green and the red fluorescence microphotographs of 10 fields of view in each experiment.

## 3. Results

### 3.1. Antimicrobial Activity against Planktonic Cells

In our previous works we have reported the synthesis of quaternary ammonium and phosphonium salts of pyridoxine and 6-hydroxymethylpyridoxine which had demonstrated activity against* S. aureus *and* S. epidermidis *[24–26]. Both strains were sensitive to ciprofloxacin according to EUCAST rules (http://mic.eucast.org/) and to miramistin, a new generation Russian antiseptic drug belonging to quaternary ammonium salts (see Table 1). An intermediate resistance to vancomycin was detected; therefore, it was out of further investigations. Ammonium (**1**,** 2**, and** 3**) and phosphonium (**4**,** 5**, and** 6**) salts of pyridoxine and 6-hydroxymethylpyridoxine exhibited MIC values a little higher than those of the reference antibiotics.

### 3.2. Antimicrobial Activity against Biofilm-Embedded and Biofilm-Detached Cells

**Figure 2:**
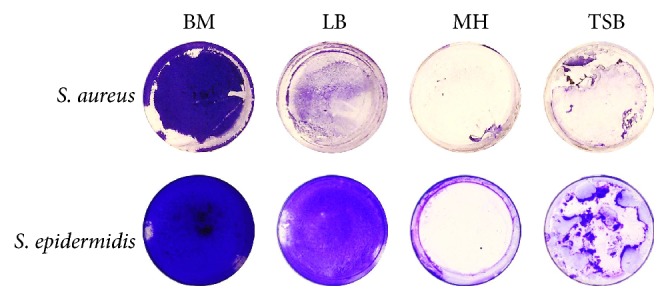
The biofilm formation by* S. aureus* and* S. epidermidis* when growing on Basal medium (BM), Luria-Bertani broth (LB), Mueller-Hinton broth (MH), or Trypticase soy broth (TSB). 72-hour-old biofilms were stained by crystal violet.

While being active against planktonic cells, many antimicrobial agents are inefficient against biofilm-embedded bacteria. We asked whether the new derivatives of quaternary ammonium and phosphonium compounds characterized by cationic groups are able to diffuse into the biofilm matrix and kill* Staphylococci* there.* S. aureus *and* S. epidermidis* were grown using 35 mm adhesive TC-treated culture plates in a BM broth that was found to provide repeatable and stable formation of rigid biofilms by both strains in 72 h, in contrast to LB, Mueller-Hinton or Trypticase soy broth (Figure 2). Next the wells were carefully washed twice by sterile BM to remove planktonic and nonadherent cells. Plates were refilled with fresh BM and the pyridoxine derivatives (ammonium and phosphonium salts) were added to the final concentrations of 4, 8, 16, 32, and 64 *µ*g/mL that correspond in average to the 1-, 2-, 4-, 8-, and 16-fold exceedance of the established MIC values against planktonic cells in BM broth (see Table 1). After 24 h of cultivation in the presence of antimicrobials, the biofilm was washed twice with PBS and the viability of detached and biofilm-embedded cells was analyzed.

**Figure 3:**
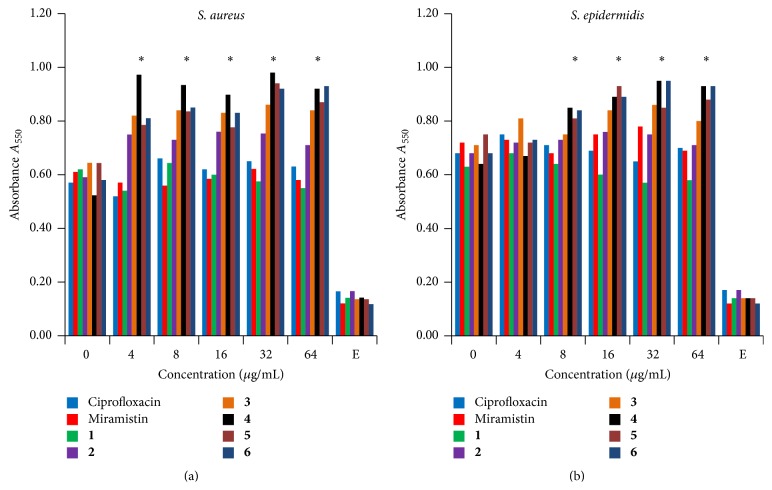
The effect of pyridoxine derivatives on the thickness of preformed biofilms of (a)* S. aureus* and (b)* S. epidermidis*. Three-day-old biofilms were washed twice by sterile broth, exposed for 24 h to antimicrobials at concentrations as indicated and then stained by crystal violet. Wells incubated with pure medium served as a control (indicated as E). Standard deviations in each case did not exceed 15% and are not provided. The values for** 3**–**6** significantly differ from the control value (without any antimicrobials) that were indicated with *P* < 0.05 as judged by Manna-Whitney test.

First, the residual biofilm was verified by crystal violet staining. Neither of the tested compounds led to any significant disruption of preformed biofilm of* Staphylococci* (Figure 3) and the broth remained almost clear (OD_600_ < 0.1) in the wells containing compounds at the concentrations of 4 *µ*g/mL and higher.

**Figure 4:**
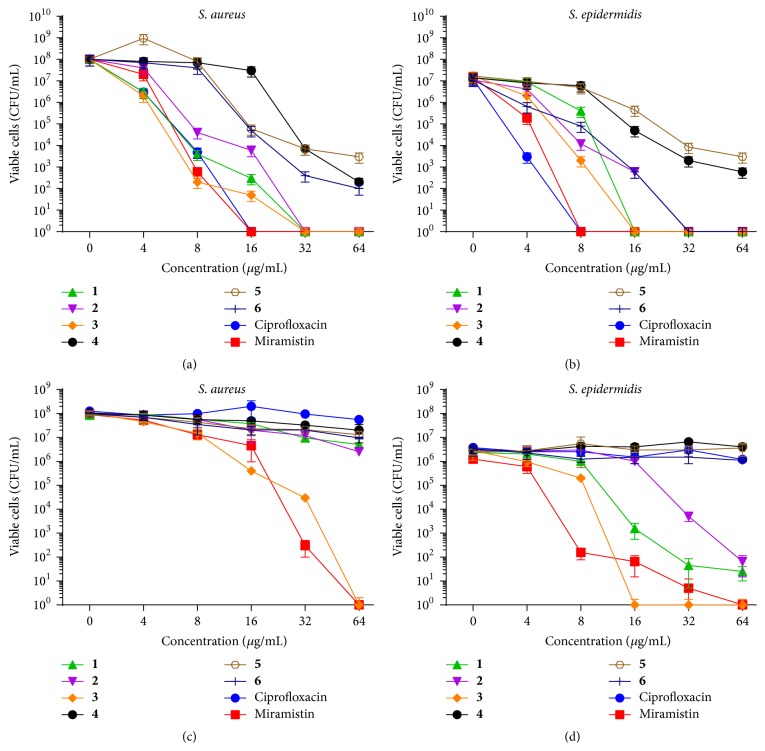
The amount of viable* S. aureus* (a, c) and* S. epidermidis* (b, d) biofilm-detached (a, b) and biofilm-embedded (c, d) cells after 24 h exposition to antimicrobials, CFUs/mL.

Despite the biofilm integrity, in the wells containing ciprofloxacin, a fluoroquinolone, at the concentrations of 16 *µ*g/mL or 8 *µ*g/mL, no viable* S. aureus *or* S. epidermidis* cells, respectively, were detected by the drop plate method in the culture liquids (Figures 4(a) and 4(b)). While similar concentrations of miramistin, a quaternary ammonium salt, were enough to kill detached* Staphylococci *cells, higher concentrations of ammonium quaternary salts** 1**–**3** (16–32 *µ*g/mL) were required for that. Among phosphonium salts, only** 6** killed detached* S. epidermidis* cells at the concentration 32 *µ*g/mL.

To evaluate the viability of the biofilm-embedded cells after 24 h treatment by antimicrobials, the washed biofilms were resuspended in PBS and analyzed by drop plate method. Neither ciprofloxacin nor pyridoxine phosphonium salts (**4**–**6**) led to significant decrease of biofilm-embedded cells (Figures 4(c) and 4(d)). MBC^adh^ for miramistin, a quaternary ammonium salt, was defined as 64 *μ*g/mL for both* S. aureus *or* S. epidermidis.* Compound** 3**, quaternary ammonium salt of pyridoxine (N,N-dimethyl-N-((2,2,8-trimethyl-4H-[1,3]dioxino[4,5-c]pyridin-5-yl)methyl)octadecan-1-aminium chloride), demonstrated MBC^adh^ of 64 *μ*g/mL* S. aureus *and 16 *μ*g/mL for* S. epidermidis*, demonstrating ultimate efficiency against biofilm-embedded* S. epidermidis* cells compared to ciprofloxacin. Notably, the viability of the cells was affected while they remained in the biofilm that sustained its integrity, a likely indication that these compounds were able to successfully penetrate into the biofilm.

### 3.3. Fluorescent Microscopy

**Figure 5:**
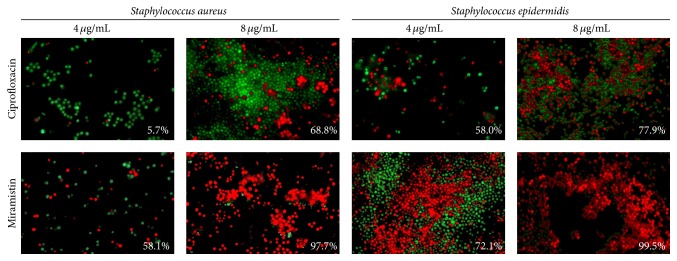
The antibacterial effect of ciprofloxacin and miramistin against biofilm-embedded* S. aureus* and* S. epidermidis* cells. Bacteria were grown for 72 h to form a rigid biofilm. Next the medium was replaced by the fresh one after double washing to remove nonadherent cells; antibiotics were added as indicated followed by 24 h incubation. Afterwards the number of viable cells was evaluated by staining the cells with propidium iodide and acridine orange. The estimated percentage of nonviable cells is given in the lower right corner of each panel. Magnification ×63.

The drop plate method allowed estimating the number of viable cells but not their fraction. Therefore, to evaluate the fraction of viable biofilm-embedded cells, we used differential fluorescent staining with propidium iodide and acridine orange. The biofilm-embedded* S. aureus *cells were almost unaffected by the ciprofloxacin at concentration of 8 *µ*g/mL ((Figure 5) up to 64 *µ*g/mL, not shown), while 8 *µ*g/mL of miramistin resulted on average in the death of 97.7%* S. aureus *cells with 3.4% standard deviation (see Figure 5).

**Figure 6:**
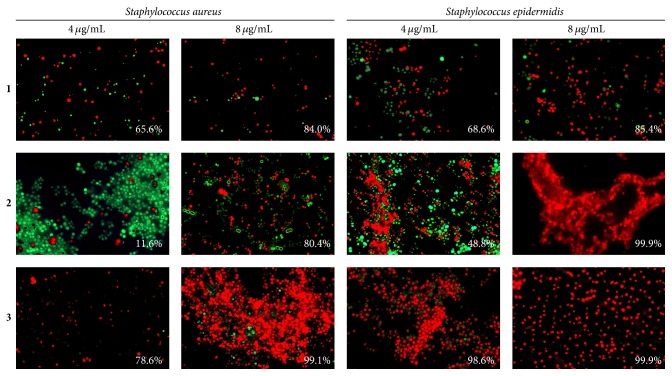
The antibacterial effect of quaternary ammonium salts of pyridoxine against biofilm-embedded* S. aureus* and* S. epidermidis* cells. Bacteria were grown for 72 h to form a rigid biofilm. Next the medium was replaced by the fresh one after double washing to remove nonadherent cells; compounds were added as indicated followed by 24 h incubation. Afterwards the number of viable cells was evaluated by staining the cells with propidium iodide and acridine orange. The estimated percentage of nonviable cells is given in the lower right corner of each panel. Magnification ×63.

Among the quaternary ammonium compounds monoammonium salt of pyridoxine** 3** led to the death of 99% of both* S. aureus *and* S. epidermidis* cells at the concentration of 8 *µ*g/mL (Figure 6). Bisammonium salt of 6-hydroxymethylpyridoxine** 2** exhibited high activity against* S. epidermidis* only, killing about 99.9% of the bacteria (with 3.5% standard deviation) at the concentration of 8 *µ*g/mL (Figure 6), while, in the presence of monoammonium salt of 6-hydroxymethylpyridoxine** 1**, only 85 ± 5.6% of the bacterial cells were identified as nonviable at the same concentration (see Figure 6). Further increase in the concentrations of quaternary ammonium compounds up to 64 *µ*g/mL did not result in any visible increase of dead cells fraction (not shown).

**Figure 7:**
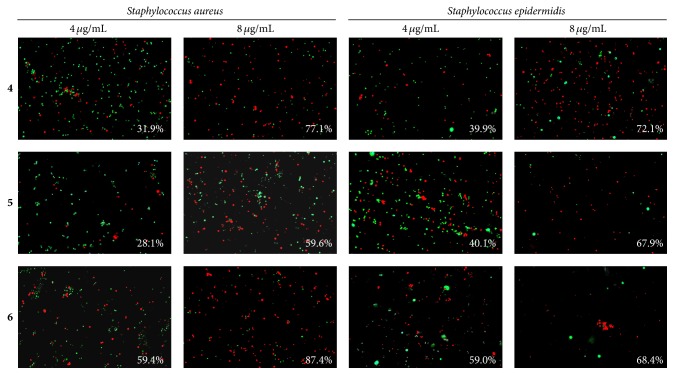
The antibacterial effect of bisphosphonium salts of pyridoxine against biofilm-embedded* S. aureus* and* S. epidermidis* cells. Bacteria were grown for 72 h to form a rigid biofilm. Next the medium was replaced by the fresh one after double washing to remove nonadherent cells; compounds were added as indicated followed by 24 h incubation. Afterwards the number of viable cells was evaluated by staining the cells with propidium iodide and acridine orange. The estimated percentage of nonviable cells is given in the lower right corner of each panel. Magnification ×40.

Surprisingly, bisphosphonium salts of 6-hydroxymethylpyridoxine demonstrated low efficiency against both* S. aureus *and* S. epidermidis* located in biofilm, despite their lower MIC values compared to the quaternary ammonium compounds against planktonic cells. In the presence of 8 *µ*g/mL of either compound** 4** or** 5** or** 6** only approximately 68–77% of the cells were identified as killed (Figure 7).

## 4. Discussion

Historically, drug discovery mainly focused on the planktonic forms of bacteria and many antibiotics targeted individual bacterial cells while remaining inefficient against microorganisms embedded in the biofilm matrix [1, 2, 34, 36].* Staphylococcus aureus *and* Staphylococcus epidermidis* often form rugged biofilms on the surfaces of medical devices, wounds, and burns this way causing healing prolongation, chronic reinfections, and inflammatory complications during the postoperative period [3–5]. While in the biofilm, bacteria are extremely resistant to biocides, antibiotics and to the human immune system [1, 2].

Our results indicate that ciprofloxacin, while being efficient against planktonic* S. aureus *and* S. epidermidis*  (MIC of 0.5 and 1 *µ*g/mL, MBC of 8 and 4 *µ*g/mL, resp.; see Table 1) and against the biofilm-detached cells (bactericidal concentration was found to be 8 and 4 *µ*g/mL, resp.; see Figures 4(a) and 4(b)), did not affect the biofilm-embedded cells (see Figures 4(c), 4(d), and 5). Similarly, the phosphonium and the ammonium salts of 6-hydroxymethylpyridoxine (compounds** 1**,** 4**–**6**) seem to be inefficient against biofilm-embedded* S. aureus *and* S. epidermidis *(Figures 4(c), 4(d), 6, and 7), although exhibiting low MICs against planktonic cell (Table 1), like many other known quaternary ammonium salts [21] and novel phosphonium salts [24, 25]. Of note, the phosphonium salts of 6-hydroxymethylpyridoxine (compounds** 4**–**6**) led to the spreading of cells in the biofilm matrix causing small number of cells in the fields of view (Figure 5). Apparently, these compounds cause the increased production of extracellular matrix and thicker biofilm as a stress response (see Figure 2).

In contrast, the quaternary ammonium salt of pyridoxine N,N-dimethyl-N-((2,2,8-trimethyl-4H-[1,3]dioxino[4,5-c]pyridin-5-yl)methyl)octadecan-1-aminium chloride (**3**) demonstrated ultimate biocidal activity against both planktonic* S. aureus *and* S. epidermidis* cells and against the cells embedded in the biofilm polysaccharide matrix (Figures 4 and 6) with MBC^adh^ found to be 64 *µ*g/mL against* S. aureus *and 16 *µ*g/mL* S. epidermidis*. Therefore we suggest that the quaternary ammonium salts of pyridoxine are more perspective in the development of antimicrobials against* Staphylococcus* compared to the 6-hydroxymethylpyridoxine derivatives. We speculate that** 3** penetrates into the biofilm matrix as has been shown previously for other quaternary ammonium salts [21], since no disruption of the biofilm could be observed in the presence of this compound (Figure 3). Since very few antibiotics that are able to penetrate themselves into biofilms are currently known [15–17], the particular mechanism of the** 3** activity against biofilm-embedded cells is discussable. We hypothesize that the large hydrophobic C_18_ chain leads to the mechanism reminiscent of the immobilization of enzymes on nonsoluble carriers like lipid and polymer nanoparticles this way leading to the increase in their antimicrobial efficacy by improved diffusion [18].

The obvious disadvantage of the pyridoxine derivatives synthetized is their cytotoxicity, although it is comparable to that one of miramistin, and thus pyridoxine derivatives require further optimization of their structure (see Table 1). Nevertheless, we suggest that quaternary ammonium salts of pyridoxine derivatives might be helpful to design new synthetic antibiotics and disinfectants for external application against biofilm-embedded cells, disinfection of noncritical surfaces, and many other applications [19, 20].

## 5. Conclusion

To conclude, the quaternary ammonium salt of pyridoxine N,N-dimethyl-N-((2,2,8-trimethyl-4H-[1,3]dioxino[4,5-c]pyridin-5-yl)methyl)octadecan-1-aminium chloride (**3**) demonstrated high biocidal activity against planktonic* S. aureus *and* S. epidermidis* and against the cells embedded in the polysaccharide matrix of the biofilm. Therefore we suggest that quaternary ammonium salts of pyridoxine derivatives might be helpful to design new synthetic antibiotics and disinfectants for external application against biofilm-embedded cells.

## Figures and Tables

**Table 1:** Minimum inhibitory concentrations (MICs, *μ*g/mL) and minimum bactericidal concentrations (MBCs, *μ*g/mL of compounds against *S*. *aureus *and *S*. *epidermidis* in Mueller-Hinton (Basal medium) broth).

Compound	*Staphylococcus aureus*		*Staphylococcus epidermidis*		CC_50_ for HEK-293 cells, *μ*g/mL [9–11]
	MIC	MBC	MIC	MBC	
Miramistin	1 (2)	8 (8)	1 (2)	4 (4)	1.5
Ciprofloxacin hydrochloride	0.5 (2)	8 (8)	1 (2)	4 (4)	ND^*∗*^
Vancomycin	4 (4)	ND	4 (4)	ND	ND
**1**	2 (4)	8 (8)	2 (4)	8 (8)	1.3
**2**	2 (4)	16 (16)	2 (4)	16 (16)	5.4
**3**	2 (4)	8 (8)	2 (4)	8 (8)	1.4
**4**	2 (2)	16 (32)	2 (2)	16 (16)	72
**5**	2 (4)	32 (32)	2 (2)	32 (32)	200
**6**	1 (1)	16 (16)	1 (1)	16 (16)	3

^*∗*^Not determined.
